# Implementation of Electronic Medical Records in Mental Health Settings: Scoping Review

**DOI:** 10.2196/30564

**Published:** 2021-09-07

**Authors:** Yvonne Zurynski, Louise A Ellis, Huong Ly Tong, Liliana Laranjo, Robyn Clay-Williams, Luke Testa, Isabelle Meulenbroeks, Charmaine Turton, Grant Sara

**Affiliations:** 1 Centre for Healthcare Resilience and Implementation Science Australian Institute of Health Innovation Macquarie University Sydney Australia; 2 National Health and Medical Research Council Partnership Centre for Health System Sustainability Australian Institute of Health Innovation Macquarie University Sydney Australia; 3 Centre for Health Informatics Australian Institute of Health Innovation Macquarie University Sydney Australia; 4 Westmead Applied Research Centre Faculty of Medicine and Health University of Sydney Sydney Australia; 5 Information for Mental Health System Information and Analytics Branch New South Wales Ministry of Health St Leonards Australia; 6 Northern Clinical School Faculty of Medicine and Health University of Sydney Sydney Australia; 7 Faculty of Medicine, Health and Human Sciences Macquarie University Sydney Australia

**Keywords:** electronic medical records, health information technology, implementation, mental health

## Abstract

**Background:**

The success of electronic medical records (EMRs) is dependent on implementation features, such as usability and fit with clinical processes. The use of EMRs in mental health settings brings additional and specific challenges owing to the personal, detailed, narrative, and exploratory nature of the assessment, diagnosis, and treatment in this field. Understanding the determinants of successful EMR implementation is imperative to guide the future design, implementation, and investment of EMRs in the mental health field.

**Objective:**

We intended to explore evidence on effective EMR implementation for mental health settings and provide recommendations to support the design, adoption, usability, and outcomes.

**Methods:**

The scoping review combined two search strategies that focused on clinician-facing EMRs, one for primary studies in mental health settings and one for reviews of peer-reviewed literature in any health setting. Three databases (Medline, EMBASE, and PsycINFO) were searched from January 2010 to June 2020 using keywords to describe EMRs, settings, and impacts. The Proctor framework for implementation outcomes was used to guide data extraction and synthesis. Constructs in this framework include adoption, acceptability, appropriateness, feasibility, fidelity, cost, penetration, and sustainability. Quality assessment was conducted using a modified Hawker appraisal tool and the Joanna Briggs Institute Critical Appraisal Checklist for Systematic Reviews and Research Syntheses.

**Results:**

This review included 23 studies, namely 12 primary studies in mental health settings and 11 reviews. Overall, the results suggested that adoption of EMRs was impacted by financial, technical, and organizational factors, as well as clinician perceptions of appropriateness and acceptability. EMRs were perceived as acceptable and appropriate by clinicians if the system did not interrupt workflow and improved documentation completeness and accuracy. Clinicians were more likely to value EMRs if they supported quality of care, were fit for purpose, did not interfere with the clinician-patient relationship, and were operated with readily available technical support. Evidence on the feasibility of the implemented EMRs was mixed; the primary studies and reviews found mixed impacts on documentation quality and time; one primary study found downward trends in adverse events, whereas a review found improvements in care quality. Five papers provided information on implementation outcomes such as cost and fidelity, and none reported on the penetration and sustainability of EMRs.

**Conclusions:**

The body of evidence relating to EMR implementation in mental health settings is limited. Implementation of EMRs could benefit from methods used in general health settings such as co-designing the software and tailoring EMRs to clinical needs and workflows to improve usability and acceptance. Studies in mental health and general health settings rarely focused on long-term implementation outcomes such as penetration and sustainability. Future evaluations of EMRs in all settings should consider long-term impacts to address current knowledge gaps.

## Introduction

Information and information transfer are critical to the delivery of health care services, including in mental health settings [1]. Modern health care increasingly relies on new information technology (IT) systems to store, retrieve, and transfer information to support decision-making for care and administrative processes [2]. Among the health-related IT systems currently in use, electronic medical records (EMRs) are the most widely implemented across many settings [3]. In their simplest form, EMRs are digital versions of case histories containing patient health–related information, but they can also support artificial intelligence capabilities, clinical decision-support systems, natural language processing, and so on [4]. EMRs have the potential to improve adherence to clinical guidelines across all settings [5], thereby reducing resource wastage, increasing care quality, and reducing patient harm. Examples include improved prescribing practices and medication safety through integrated electronic ordering systems [6] and reductions in inappropriate laboratory testing because of integrated decision-support tools [7]. Ultimately, EMRs are expected to contribute to creating safer and more effective health systems [5].

Although several studies identifying the potential of EMRs have been published, evidence on their benefit to organizational, clinical, and patient outcomes after implementation continues to be mixed, with success appearing to be largely dependent on the design and fit with the local health care settings and workflows. For example, implementation of the same EMR system in two different university hospitals revealed that the time spent on documentation increased in one site but decreased in the other [8]. Furthermore, high-profile, unintended consequences because of EMR implementation by-products have been reported in recent times. A notable example includes the implementation of a £200 million EMR system in a major UK teaching hospital, leading to reduced performance and demoralized staff [9]. Poor usability of EMRs can impact quality of care and patient safety, as poor fit and design may cause fatigue, delayed case note entry, and adjacency errors [10]. As research on the implementation of EMRs continues to emerge, there is a strong need to understand the processes, systems, contexts, and human factors that influence successful implementation [11].

Although the adoption of EMRs has grown significantly in recent years, research that is specific to mental health settings or mental health clinicians has been minimal. Documentation in mental health settings brings unique challenges for the implementation of EMRs. Effective mental health documentation requires the recording of individualized, detailed, and narrative information, which is not easily reduced to checklists [12]. Care is often long term and multidisciplinary, requiring staff of different disciplines to record and retrieve information over long periods or in different settings (eg, hospital and community). Hence, the implementation of EMRs in mental health settings may have specific negative impacts, either real or perceived, on patient-centered care, the ability to develop the patient-clinician rapport, and on clinician time. Understanding the available evidence on implementation determinants and outcomes of EMRs in mental health settings, as well as the implementation features that contribute to its success or failure, could aid the future investment, design, and implementation of EMRs in this field.

The aim of this scoping review of the peer-reviewed literature was to provide a synthesis of implementation studies relevant to EMRs in mental health settings and inform EMR mental health policy recommendations in New South Wales, Australia. To provide in-depth recommendations, the review also considered broader evidence from general health settings to reflect on EMR implementation lessons. The specific objectives of this scoping review were to (1) identify published studies pertaining to the implementation of EMRs in mental health settings and literature reviews in general health settings, (2) synthesize the specific implementation determinants and outcomes examined in these studies according to the Proctor framework for implementation outcomes [13], and (3) provide local policy recommendations for future design and implementation of EMRs in mental health settings based on the findings.

## Methods

### Review Protocol

Our scoping review followed a predetermined (but unregistered) protocol that was developed in accordance with the PRISMA-ScR (Preferred Reporting Items of Systematic Review and Meta-Analyses Extension for Scoping Reviews) [14,15] and followed methods used in published peer-reviewed scoping reviews [16]. An exploratory search of 1 database over a 2-year period, conducted in consultation with a medical librarian and mental health experts on our team (GS and CT), confirmed that the studies on EMRs implemented in mental health settings were limited. Therefore, in our scoping review, we also conducted a review of reviews to capture implementation literature across EMRs in all health settings and not just mental health, given that the broad issues around the usability of EMRs in general health settings are potentially relevant. The results of both search strategies were analyzed; for synthesis, we used a combination of results from primary studies and review papers.

In our scoping review, we defined mental health professionals as psychiatrists, psychologists, nurses, and any other health professional involved in treating people with mental health disorders in health service settings, including allied health professionals. These settings could be mental health clinics, or general inpatient or outpatient clinics but needed to be in high-income countries. High-income countries were classified as category 1 countries by the Organisation for Economic Co-operation and Development (OECD) [17]. This criterion was used to maintain relevance to the local policy setting context. Implementation determinants were defined as barriers and enablers that may prevent or facilitate improvements in practice [18], as reported in the included studies. The Proctor framework provides a systematic taxonomy of implementation outcomes (ie, acceptability, adoption, appropriateness, feasibility, fidelity, implementation cost, penetration, and sustainability), distinguishing these from service and patient outcomes [13].

### Search Strategy

Our scoping review combined two systematic searches; the first captured published studies reporting primary data on the use and implementation of clinician-facing EMRs specifically in mental health settings (henceforth termed “primary studies”). The second search captured published reviews on the use of clinician-facing EMRs as implemented in all health settings irrespective of their relationship to mental health (henceforth termed “reviews”). The searches were conducted in three academic databases (MEDLINE via the PubMed Interface, EMBASE, and PsycINFO) and used the terms outlined in Table 1. Additionally, we manually searched the reference lists of the included studies (primary studies and reviews) for other relevant publications. All searches were limited to studies and reviews published between January 2010 and June 2020. The search strategies were devised by the review team with the assistance of an experienced medical librarian.

**Table 1:** Database search strategy used in MEDLINE.

Construct	Search terms for primary studies		Search terms for reviews	
EMR^a^-related terms	“Electronic Health Records”[MeSH] OR Medical Records Systems, Computerized [MeSH] OR ((health record*^b^ OR medical record* OR healthcare record* OR health care record* OR clinical record*) AND (digital OR electronic OR computerized OR computerized OR ambulatory)) [Title/Abstract]		“Electronic Health Records”[MeSH] OR Medical Records Systems, Computerized [MeSH] OR ((health record* OR medical record* OR healthcare record* OR health care record* OR clinical record*) AND (digital OR electronic OR computerized OR computerized OR ambulatory)) [Title/Abstract]	
Health professional–related terms	“Psychiatry”[MeSH] OR “Psychiatric Nursing”[MeSH] OR (mental health OR psychiatric nurs* OR psychiatry OR psychiatrist OR psychology OR psychologist) [Title/Abstract]		“Psychiatry”[MeSH] OR “Psychiatric Nursing”[MeSH] OR “Physicians”[MeSH] OR “Nurses”[MeSH] OR “Health Personnel”[MeSH] OR (Physician OR nurse OR doctor OR psychiatrist OR psychologist OR health professional OR health personnel OR psychiatric nursing) [Title/Abstract]	
Impact-related terms	(uptake OR adoption OR usability OR utility OR utilization OR utilization OR evaluate OR evaluation OR implementation OR acceptance OR acceptability) [Title/Abstract]	(uptake OR adoption OR usability OR utility OR utilization OR utilization OR evaluate OR evaluation OR implementation OR acceptance OR acceptability) [Title/Abstract]		
Additional limiters	Published in English AND published between January 2010 and June 2020			(Systematic review or meta-analysis) AND published in English AND published between January 2010 and June 2020

^a^EMR: electronic medical record.

^b^Asterisk indicates truncation.

### Inclusion and Exclusion Criteria

In both searches, articles were included if they met the following inclusion criteria: investigated implemented clinician-facing EMRs; conducted in high-income countries (countries classified as category 1 by the OECD [17]); assessed and reported implementation outcomes and contextual determinants of implementation (ie, barriers and facilitators); and published between January 1, 2010, and June 30, 2020. The population and study type inclusion criteria differed between the searches. The review of primary studies included studies related to mental health clinicians, whereas the review of reviews included literature reviews of studies about any health professionals in any health setting.

In both searches, articles were excluded if the implemented EMRs were exclusively patient-facing ones, they did not report on implementation processes or outcomes, or they were not published in English. The complete list of the inclusion and exclusion criteria is available in Multimedia Appendix 1.

### Screening, Data Extraction, and Synthesis Procedures

Reference details, including abstracts, were downloaded into the reference management software EndNote X8 (Clarivate) [19]; duplicates were removed and the deduplicated list was exported to Rayyan QCRI [20], a systematic reviews web app, for title and abstract screening. Five investigators (HLT, LT, LAE, AG, and IM) independently conducted the two-phase screening process: (1) title and abstract screening and (2) full-text screening. Two investigators (HLT and IM) cross-checked 50% of the records to ensure that article screening was consistent in accordance with accepted practices [21]. Interrater reliability (Cohen kappa coefficient) in this cross-checking indicated strong agreement (>0.8) [22]. A custom data extraction workbook in Excel (Microsoft Corporation) was developed and tested. Data were systematically extracted by six investigators (HLT, LL, LT, AG, LAE, and IM). Four investigators (LT, LAE, YZ, and IM) examined the data for consistency and cross-checked the extracted data against original articles.

Key information extracted included the study publication details (authors, date of publication, country of study, and number of studies in reviews), health settings, study methods (quantitative, qualitative, and mixed methods), design features of EMRs, and implementation barriers, enablers, and outcomes. To ensure consistency in our review, the Proctor framework of implementation outcomes presented in Table 2 was used as the guiding structure, with definitions tailored to suit the EMR implementation context [13].

**Table 2:** Proctor implementation outcomes as applied in this study.

Domain	Definition
Adoption	Uptake of the EMR^a^ from the professionals, organizations, and settings
Acceptability	Clinician satisfaction with various aspects of the innovation (eg, content, complexity, comfort, delivery, and credibility)
Appropriateness	Perceived fit, relevance, compatibility, suitability, usefulness, and practicability defined by clinicians
Feasibility	Actual fit or usefulness, suitability for everyday use, and practicability assessed at the level of the health service provider/organization/setting
Fidelity	Program delivered as intended, adherence by clinicians, integrity, and quality of program delivery
Cost	Financial impact of technology implementation on the health provider or organization
Penetration	Spread or reach of the technology assessed at the organization or setting level
Sustainability	Maintenance or integration of a technology within a health service

^a^EMR: electronic medical record.

### Assessment of Evidence Quality

Primary studies were appraised for quality using a modified Hawker appraisal tool and scoring system [23,24]. This tool was selected as it is designed to review evidence from a variety of methods [23]. The Critical Appraisal Checklist for Systematic Reviews and Research Syntheses developed by the Joanna Briggs Institute was used to appraise the systematic review studies [25]. Two investigators (HLT and IM) appraised 10% of the articles independently to ensure consistency. Quality assessment results were reported to reflect the quality of the studies and reviews included in our scoping review. We did not exclude studies based on quality assessment.

### Data Analysis and Synthesis

The extracted data were analyzed for common features and summarized into tables. Implementation outcomes were grouped by outcomes (eg, satisfaction), and barriers and enablers were grouped by themes (eg, technical factors). Barriers, enablers, and implementation outcomes were also categorized using the Proctor framework (Table 2), while recognizing some degree of overlap among constructs as suggested by other publications [13,26]. Assignments to the constructs were based on the definitions applied by the review team (Table 2) rather than the assignment made by the authors of the included articles owing to inconsistencies in the manner of defining, measuring, and reporting implementation outcomes [13]. Three investigators (YZ, LAE, and IM) reviewed the assignment of all the results, and any discrepancies were discussed among the three authors until a consensus was reached. Summary statistics (frequencies and proportions) were calculated for the final assignment.

## Results

### Search Results and Study Selection

**Figure 1:**
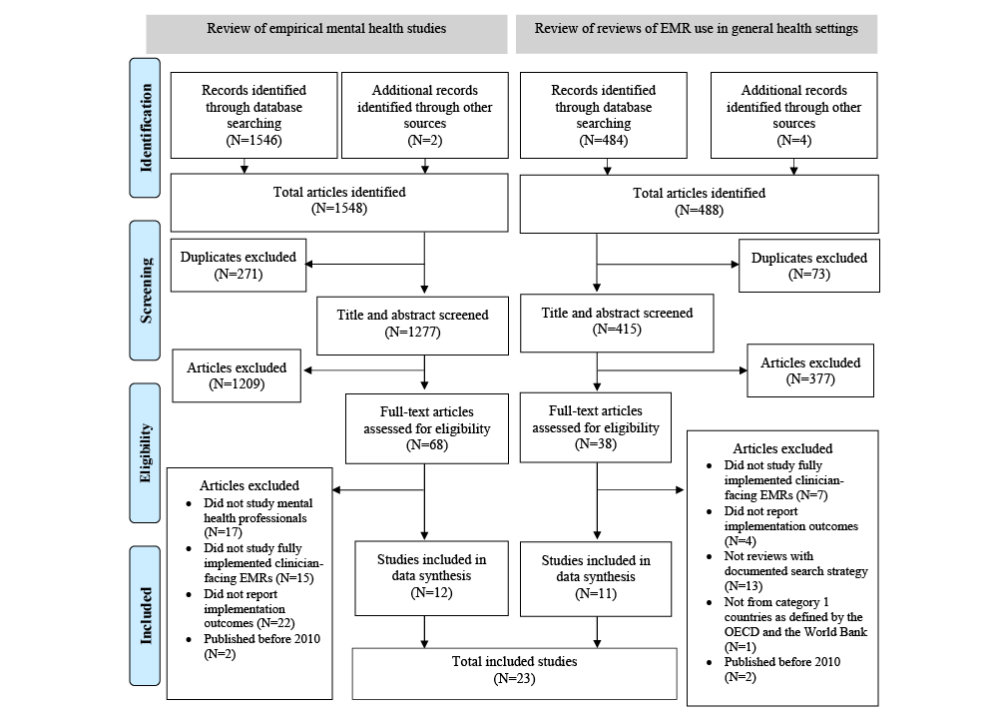
Selection of primary studies and reviews. EMR: electronic medical record; OECD: Organisation for Economic Co-operation and Development.

The search for primary studies yielded 1546 results relevant to mental health professionals or settings (Medline: 606; EMBASE: 620; PsycINFO: 320). Manually searching the article reference lists yielded 2 more papers. Among these, 271 duplicates were removed; after title/abstract screening, 1209 papers were excluded because they did not meet the inclusion and exclusion criteria. Furthermore, 68 studies underwent full-text review, and another 56 papers were excluded. We included 12 primary studies for data extraction and synthesis, as shown in Figure 1.

The search for reviews yielded 484 results (Medline: 175; EMBASE: 297; PsycINFO: 12). We identified 4 additional papers by manually searching article reference lists. Then, 73 duplicates were removed, and after title and abstract screening, 377 papers were excluded. Another 27 were excluded after full-text review, and 11 were included for data extraction and synthesis (Figure 1). A total of 23 studies were included for data extraction and synthesis from the 2 searches.

Half of the primary studies (6/12, 50%) were from the United States of America. The remaining primary studies were from Canada (2/12, 16.7%), the United Kingdom (2/12, 16.7%), France (1/12, 8.3%), and Sweden (1/12, 8.3%), as shown in Table 3. Most primary studies were conducted using quantitative methodologies (5/12, 42%), and fewer studies were conducted using qualitative (3/12, 25%) or mixed (4/12, 33%) methodologies. Each of the review studies included publications from several countries; however, in each review, at least more than 50% of the countries were OECD nations, as observed in Table 4. Among these 11 review studies, 1 (9%) focused on mental health settings [27], and the remaining 10 (91%) involved general health settings.

**Table 3:** Summary of the included primary studies.

Study	Country	Setting	EMR^a^ implemented	Participants
Boyer et al [28]	France	Psychiatric hospital	Hospital EMR including coded data, unstructured text, and scanned paper documents	115 health professionals
Bruns et al [29]	United States	Mental health facilities	EMR with standardization of information, assessments, and diagnosis; facilitated a coordinated care plan, team communication, and routine reporting	34 wraparound care coordinators
Erlingsdóttir et al [30]	Sweden	Psychiatry services	Patient-accessible EMR	871 mental health professionals preimplementation; 699 postimplementation
Golberstein et al [31]	United States	Primary care clinics	EMR prompting specific mental health questions and enabling e-consult ordering with psychiatry	Primary care providers (457 in the first wave; 499 in the second) from 45 clinics
Jetelina et al [32]	United States	Primary care	EMR with referral pathways, screening tools (point-and-click tools, drop-down menus, auto calculators, and auto population of some fields), and tracking and documentation of clinical and social information and goal setting	6 community care clinics with a mix of primary care, and psychology and social work
Madden et al [33]	United States	Medical practice	Not specified	Health insurance plan members with depression (5140), bipolar disorder (462), and a control group (43,582)
Martin et al [34]	Canada	Psychiatric hospital	Not specified	24 nurses
Reyes-Portillo et al [35]	United States	Child and youth psychiatry clinic	Alert in existing EMR that triggered a safety plan when a suicidal ideation, a plan, or an attempt was recorded	40 mental health clinicians
Riahi et al [36]	Canada	Mental health facility	EMR containing closed-loop medication administration, assessment and screening tools, care plan, details of restraint and seclusion, clinical practice guidelines, and infection control details	1300 facility staff
Ser et al [37]	United Kingdom	Mental health hospitals	Interoperable EMR	33 hospital staff
Skelton et al [38]	United Kingdom	Older adult psychiatric inpatient ward	Out-of-hours handover built into existing EMR	10 doctors
Stanhope et al [39]	United States	Community mental health clinics	Delivering person-centered care in the context of different EMRs	31 clinical supervisors and 52 direct care staff

^a^EMR: electronic medical record.

**Table 4:** Summary of the included reviews.

Study	Country (number of studies)	Setting	EMR^a^ implemented	Included studies, n
Baumann et al [40]	United States (12), Australia (5), Germany (5), United Kingdom (1), Canada (1), Austria (1), Denmark (1), Greece (1), and France (1)	Academic, private, and community hospitals	Not specified	28
Boonstra et al [41]	United States (17), Canada (2), Norway (1), and Ireland (1)	General health settings	Not specified	22
Castillo et al [42]	United States (52), Canada (4), Australia (3), Germany (2), International group (1), Denmark (1), France (1), Sweden (1), Hong Kong (1), United Kingdom (1), and Norway (1)	General health settings	Not specified	68
Delardes et al [43]	United Kingdom (4), United States (3), Ireland (1), and Taiwan (1)	General health settings	Not specified	9
Gephart et al [44]	United States (4) and Sweden (1)	General health settings	Not specified	5
Goldstein et al [45]	United States (8), Austria (1), Brazil (1), Canada (1), and Switzerland (1)	General health settings	Not specified	12
Goldzweig et al [46]	United States (20), France (1), Canada (1), and Austria (1)	Academic medical centers	Classification of radiology ordering of EMR interventions into four categories: (1) display of information, (2) patients’ clinical information linked with recommendations, (3) soft stop if order contradicts recommendations, and (4) hard-stop software preventing inappropriate ordering	23
Lau et al [47]	United States (11), United Kingdom (10), the Netherlands (5), Canada (4), Australia (4), Norway (2), and New Zealand (2)	General health settings	Not specified	43
Meißner and Schnepp [48]	United States (4) and Australia (3)	Residential aged care facilities	Not specified	7
Nguyen et al [49]	United States (62), Denmark (5), England (5), Norway (4), Canada (3), Sweden (1), Australia (2), the Netherlands (2), Ireland (2), Israel (2), Austria (1), Cyprus (1), France (1), Serbia (1), Sweden (1), Japan (1), Korea (1), Kuwait (1), Cameroon (1), and Uganda (1)	General health settings	Not specified	98
Strudwick and Eyasu [27]	Germany (1), England (2), France (1), Finland (1), United States (1), and Sweden (1)	Mental health/ psychiatric clinic settings	Not specified	7

^a^EMR: electronic medical record.

### Quality Assessment

The primary studies scored highly on the modified Hawker appraisal tool [23], with an average score of 30.3 (SD 3.81) out of a possible 36. The reviews scored an average of 7.6 (SD 1.45) out of a possible score of 11 on the Joanna Briggs Institute Critical Appraisal Checklist for Systematic Reviews and Research Syntheses [25] (see Table S1 of Multimedia Appendix 2 for details).

### Features of Implemented EMRs

The features of the EMRs were described in 8 of the 12 (66.7%) primary studies in mental health settings. Features ranged from the simple electronic storage of personal and health information documentation [28], e-ordering of consultations [31], and capability to enter free-text notes [30] to features that aimed to improve care quality including embedded assessment tools [32,35,36], and care coordination plans [29,32,38]. Specific examples included implementing automated alerts to develop safety plans for children and youth with suicidal ideations [35] and embedding an e-consultation pathway prompt linking primary health providers with a psychiatrist [31]. Overall, the description of EMR features was limited among the included studies. Four studies did not report on specific features; instead, they simply described the EMRs as storage of clinical notes and test results to improve the accuracy and completeness of clinical information [33,34,37,39]. Only 1 of the 11 (9.1%) reviews provided a comprehensive description of EMRs among the included studies [46] (Table 4).

### Implementation Outcomes and Determinants

#### Adoption

**Figure 2:**
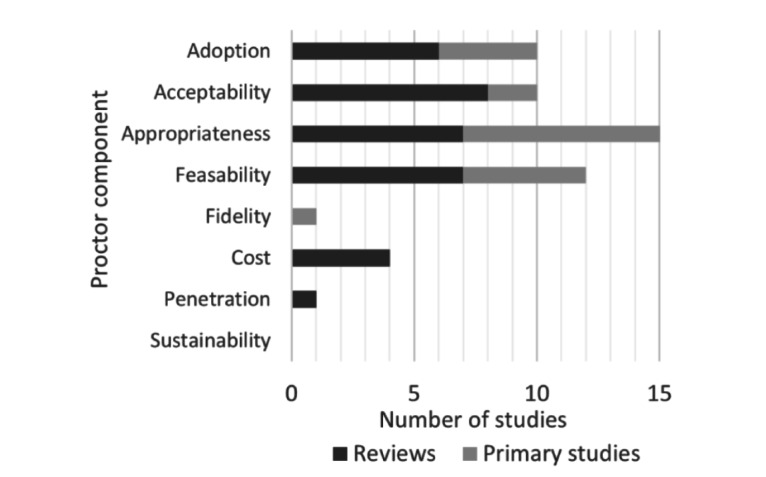
Implementation outcomes and determinants in primary studies and reviews.

Adoption was reported in 10 of the 23 included studies (43.5%), namely 4 of the 12 primary studies (33.3%) [29,32,33,38] and 6 of the 11 reviews (54.5%) [27,41,42,45,48,49], as shown in Figure 2. Factors influencing the adoption of EMRs fell into three categories: organizational, technical, and financial.

First, in the primary studies, high adoption rates were attributed to organizational support and prioritization [29], strong leadership and buy-in, greater capacity and willingness to change, engagement of staff, and formal training [32]. In contrast, poor leadership and buy-in, high staff turnover, and poor capacity or unwillingness to change, resulted in lower adoption rates [32]. Two primary studies suggested that adoption was high without reflecting on the reasons for this [29,38]. Similarly, the reviews reported that organizational structure, readiness for change, participation of leaders and end users in planning and implementation, and support for end users impacted adoption [41,42,49]. Specifically, adoption was facilitated by training [49], larger facility sizes [41,45], clinical champions/leaders [41,49], and the removal of all paper-based notes [49]. A lack of clear implementation plans was identified as an important limitation to adoption [49].

In 6 of the 11 (54.5%) reviews, adoption was reported to be limited by the technological functions and design of EMRs such as perceived limited functionality [41,45,48,49], interoperability [41,42,45,49], lack of technical support, limited clinician technical skills (real or perceived) [27,41,42,45,49], insufficient hardware [41,45,49], and system failures (software or hardware breakdowns, errors, and need for frequent rebooting) [49]. None of the primary studies reflected on technological factors influencing adoption.

One review concluded that the start-up financial cost of EMRs were the second most common barrier to adoption, after technical issues [45]. Three other reviews also cited high start-up costs as a barrier to adoption [41,45,49]. None of the primary studies reported on cost factors influencing adoption.

#### Acceptability

Among the included studies, 2 out of the 12 primary studies (16.7%) [35,38] and 8 out of the 11 reviews (72.7%) [27,41,42,44-46,48,49] reported on clinician acceptance of (or satisfaction with) the implemented EMRs or aspects of EMRs (Figure 2). Reviews reported that positive clinician attitudes were necessary for successful adoption of EMRs [42,45,48]. However, clinician satisfaction with EMRs varied in primary studies and reviews.

One primary study found that mental health clinicians were neutral about the addition of an alert for a mental health safety plan in EMRs [35], whereas another found there were fewer complaints regarding the quality of clinical handover following the introduction of out-of-hours electronic handover systems in the EMRs [38].

In reviews, poor clinician satisfaction was associated with perceptions that the new software was complex, it took time to learn and use, and that time could be used for patient care [41,45,46,49]. There were also concerns regarding patient data privacy [27,41,44,45,48]. Rigidly designed EMRs and exclusion of end users from design processes [27,41,44], poor trust in the quality of EMR vendors [41], the perception that the software demanded excessive detail [49], and previous negative experiences or negative beliefs about the usefulness of EMRs were additional important barriers to acceptability [41,49]. Likewise, one review identified that high satisfaction was associated with the perceived reliability and usability of EMRs, and adequate support for end users [49].

#### Appropriateness

The perceived appropriateness of EMRs was reported in 7 of the 11 reviews (63.6%) and 8 of the 12 primary studies, as shown in Figure 2. Clinicians often assessed EMRs as appropriate or inappropriate based on their perceived impact on the clinical workflow and productivity, quality of clinical documentation, quality of care, and patient-clinician relationships.

In the primary studies, mental health clinicians perceived EMRs as appropriate when access to documentation improved [28], the time needed to send reminders to patients decreased [29], administration time decreased [29], and time was saved on documenting follow-up appointments owing to prefilled data [32]. On the other hand, mental health clinicians believed that EMRs lacked appropriateness when workflows were blocked or slowed [28], and when clinicians needed to take additional time to design workarounds for EMRs that did not meet their needs [37,39]. Similarly, in the reviews, EMRs reportedly lacked appropriateness when documentation time increased [41], at least temporarily [48], whereas other reviews found EMRs to be appropriate when access to documentation improved [27], and when there was minimal impact on documentation time [49]. One review further identified that although EMRs saved documentation time, the standard forms were not always appropriate for documenting assessments, treatments, and goals for patients receiving mental health care [27].

In the reviews, perceptions of improved documentation quality in terms of legibility, accuracy, completeness, and consistency were associated with clinicians’ views that EMRs were appropriate [44,48,49]. However, some clinicians in mental health settings [27,30] and in general health settings [44,49], believed that EMRs lacked appropriateness owing to the requirement of excessive or redundant information or when access to patient notes became a “watered-down” version of free-text clinical notes that lacked detail [30].

Across the primary studies and reviews, EMRs were perceived as appropriate if they were also perceived to be effective in terms of improving patient care [38] through supported decision-making based on availability of up-to-date information [30,31,48,49], better team communication, and averted potential medication errors [48,49].

One review suggested that EMRs improved patient-clinician interactions owing to the accessibility of information to clinicians [49]. Other reviews found EMRs that impacted decision-making processes and workflows left clinicians feeling devalued in their clinical role and were hence considered inappropriate [42,45]. No primary studies discussed the acceptability and impact of EMRs on the patient-clinician relationship.

#### Feasibility

Feasibility of the EMRs or EMR components was investigated in 5 out of the 12 primary studies (41.7%) [33-36,38] and 7 out of the 11 reviews (63.6%) [27,40,41,43,46,47,49] (Figure 2). Across all studies, the feasibility of implementation and use of EMRs by clinicians was assessed through proxies such as documentation outcomes (time taken and completeness), frequency of adverse events, quality of care, and face-to-face clinical time. These outcome measures differ from those used under adoption, acceptability, and appropriateness, as these include quantifiable impacts of the implemented EMRs or the actual fit for purpose rather than perceptions or opinions.

In the reviews, the measured impacts included improved documentation time, as mentioned in one review involving mental health settings [27], whereas others found no difference [40,41]. Documentation time was not quantified in any of the primary studies.

Completeness of documentation varied, with one review reporting increased completeness [49], whereas another one found no impact [43]. In the primary studies, an alert system increased the number of completed mental health safety plans [36] and reduced the amount of missing data [34]. However, another primary study found that events (eg, emergency department and hospital visits or mental health diagnoses and related procedures) for mental health patients were less likely to be recorded in EMRs compared with other types of patients [33].

The impact on patient outcomes was rarely reported. A primary study found that an electronic handover system was associated with a downward trend in adverse events, but this was not statistically significant [38]. A review found that EMRs had no impact or had a small impact on adverse events such as hospital readmission [43].

Impacts of EMRs on care quality in mental health settings were not reported. However, reviews reported that EMRs reduced the time from orders to procedures [43], decreased medication errors [47], and improved appropriate ordering of radiographic tests, although they increased the number of missed tests [46].

#### Cost

None of the primary studies assessed the cost-effectiveness of EMRs. However, 4 of the 11 reviews identified that cost was a barrier to adoption [41,42,49], and interoperability of EMRs could improve long-term costs [45]; there was no evidence that costs decreased owing to improved administrative effectiveness [49]. In addition, the ongoing costs of maintaining and upgrading EMRs were reported to be high and the return on investment uncertain [41].

#### Fidelity, Penetration, and Sustainability

These domains were seldom addressed across all the 23 included studies. One primary study reported improved patient-centered care, which was one of the intended impacts (fidelity) of that specific EMR system [39], and a review reported that the rate of EMR usage across clinical settings was exceedingly slow [49]. No study addressed the sustainability of the implemented EMRs.

## Discussion

### Principal Findings

In mental health settings, the adoption of EMRs is seemingly impacted by technical and organizational factors, as well as by clinician perceptions of appropriateness and acceptability. Clinicians perceived EMRs as acceptable and appropriate if they improved documentation completeness without interrupting workflow. Clinicians tend to value EMRs that support quality of care, are fit for purpose, have readily available technical support, and do not interfere with the clinician-patient relationship. Overall, the body of evidence specific to mental health was small. The implementation determinants and outcomes identified in general health settings aligned with and expanded on the mental health–specific findings. For example, the cost of implementation was identified as an additional barrier to adoption, apart from the technical and organizational factors identified in the mental health literature. However, evidence from general health settings did not consider the unique challenges of implementing EMRs in mental health settings. We have drawn on the evidence from general and mental health settings to make three recommendations for future implementation of EMRs in mental health settings.

Firstly, EMR implementation requires embedded long-term evaluation. In this review, we identified that approximately half of the studies focused on the early‐to-middle stage implementation outcomes (ie, adoption, acceptability, appropriateness, and feasibility) [13], whereas later‐stage implementation outcomes (ie, penetration and sustainability) [13] and implementation costs were rarely evaluated. Fidelity was assessed in only one primary study in a mental health setting. This is in contrast with the implementation research outside of research on EMRs, where implementation fidelity has more often been assessed compared with other outcomes [13]. This may be related to the nature of EMR technologies, which can be tailored and used flexibly to suit particular practices or service needs [50]. Sustainability was also not reported in any of the included studies, a finding that is consistent with implementation research outside of the EMR field where the assessment of program sustainability has been identified as a neglected area [13,51,52]. Limited research on the cost, fidelity, penetration, and sustainability of EMRs suggests limited evaluation and impact assessment, and the lack of long-term goal setting, particularly at the organizational level. We recommend that future implementation of EMRs in mental health settings must include continuous and embedded evaluation to explore long-term outcomes and impacts for health professionals and patients while identifying the determinants of cost, fidelity, penetration, and sustainability. Findings from thorough evaluations are needed to inform the future design, policies, and uptake of EMRs in mental health and other health settings.

Secondly, implementation of EMRs needs to adopt co-design principles and a human factors approach, including clinician participation in formative and summative usability testing prior to and during implementation [53,54]. The successful uptake of EMRs is influenced by clinicians’ perceptions of appropriateness and acceptability. In mental health settings, this was negatively impacted when EMRs misaligned with established workflows. It was also affected by organizational factors such as high staff turnover, low staff buy-in, and low capacity or willingness to change shown by clinicians. Evidence from general health settings suggests that these determinants can be modified by specific facilitating features such as staff training, clinical champions, buy-in from clinicians and leaders, IT support, and, above all, good fit for purpose with minimal disruption to clinical workflows. However, EMRs are commonly designed by IT professionals; although well intentioned, the software is often insufficiently flexible to meet the needs of clinicians at the frontlines of care [55]. Good fit with clinical workflows and local clinical contexts can be achieved through user-centered design processes and collaboration between clinicians and IT professionals [56,57]. Outside of this review and in general health settings, authors have recommended routine use of co-design principles and frameworks, formative evaluations in consultation with clinicians, and frameworks to assess the fit of off-the-shelf EMRs [58]. In the mental health–specific literature covered in this review, co-designing was not analyzed. In future, to enhance the fit of EMRs to the unique and sensitive clinical work undertaken in mental health, we recommend that the development and implementation of EMRs include co-design and formative evaluations to achieve an optimal fit to support usability for clinicians and patient centeredness.

Lastly, the implementation of EMRs needs to be guided by theories and frameworks to successfully navigate behavior change, and interactions between people and technology. In an environment where sensitive issues are addressed and building rapport and trust with patients is especially important, simply “injecting technology” is unlikely to yield better care, experience for health professionals, or successful implementation. For example, in this review, organizational factors such as leadership and culture were the common determinants of EMR implementation [32,41,42]. It is also likely that external factors (eg, health system structure, funding, and governance) impact EMR implementation as seen in other areas of mental health [59]; however, this was not addressed in the included studies. Successful implementation of EMRs requires structured methodology and careful planning, as changes in a social environment often require new skills and can have unpredictable impacts [60]. We recommend that the development, planning, implementation, and evaluation of EMRs could be improved by applying appropriately structured guiding theories and frameworks (eg, behavior change theory [61] or the normalization process theory [62]).

### Strengths and Limitations

Despite a rigorous search strategy, it is possible that some potentially relevant studies were missed owing to a wide range of terms used to describe EMRs (eg, health information systems and electronic health records). Nevertheless, our search strategy identified over 2000 potential publications across the two search strategies, reflecting its high level of comprehensiveness.

Further, the inconsistent use of implementation outcome terminologies across the literature and some degree of overlap among constructs as suggested by other publications [13,26] made it challenging at times to classify outcomes into the Proctor categories. Although this may have resulted in the misclassification of some findings, they were applied as closely as possible to the Proctor definitions. A robust process where classifications were reviewed by three of the authors (YZ, LAE, and IM) and any discrepancies were discussed until a consensus was reached is a methodological strength supporting our synthesis.

Lastly, although we assessed study quality using validated tools, owing to the limited evidence available, it was not feasible to exclude studies or distinguish findings based on quality. Quality assessment results are described in Table S2 of Multimedia Appendix 2.

### Conclusion

The body of evidence about the implementation of EMRs in mental health settings is currently limited. Key enablers of the adoption of EMRs by clinicians in all health settings included clinician buy-in, staff training, IT support, and appropriate fit with the clinical context and workflows. Specific issues identified in mental health settings included limited suitability of the drop-down or checklist options and their impact on clinical workflows and patient-clinician interactions. Future implementation of EMRs could be facilitated through co-design with clinician end users, embedding routine implementation process evaluations, and including routine feedback from clinicians to facilitate adjustments and ensure usability and the best fit with the clinical context and person-centered care. Additionally, it is imperative that future implementations include embedded evaluations to assess long-term impacts on organizations, clinicians, and patients in mental health settings to inform future design, implementation, policy, and funding decisions. Lastly, the implementation of EMRs needs to recognize and address the interplay between the social factors and technical aspects of EMRs as a sociotechnical system to support successful uptake. Future research should consider the application of guiding social theories, implementation frameworks, and consistent use of terminology.

## Appendix

Multimedia Appendix 1 Inclusion and exclusion criteria.

Multimedia Appendix 2 Quality appraisal results of primary studies and reviews.

## Abbreviations

EMR: electronic medical record
IT: information technology
OECD: Organisation for Economic Co-operation and Development
