# Supplementary material for: Implementation of Electronic Medical Records in Mental Health Settings: Scoping Review
Source: JMIR Ment Health. 2021 Sep 7;8(9):e30564. doi: 10.2196/30564 (PMC8456340; doi:10.2196/30564)
Supplement: Multimedia Appendix 1 [file mental_v8i9e30564_app1.docx]

## Appendix 1

**Table 1.** Inclusion and exclusion criteria.

|  | Inclusion | Exclusion | R1^a^ | R2^b^ |
| --- | --- | --- | --- | --- |
| Setting | Countries that are both (1) OECD member countries and (2) category 1 countries, as defined by the OECD | Other countries | ✓ | ✓ |
| Timeframe | The last 10 years ie, January 1, 2010 to June 30, 2020 | Before 2010 | ✓ | ✓ |
| Language | English only | Other languages | ✓ | ✓ |
| Population | Clinicians/ health professionals (eg, doctors/physicians, nurses) | Exclude studies focusing on   - patients only - pharmacists - medical students |  | X |
|  | Mental health clinicians (eg, psychiatrists, mental health nurses) or any other clinicians working in a mental health setting | Other clinicians | ✓ |  |
| Intervention | Clinician-facing electronic medical records (EMRs) that have been implemented and used in health settings  Inclusion of EMRs used by clinicians and patients | - Patient-facing EMRs only - Studies where an EMR was not implemented | ✓ | ✓ |
| Comparison | Any |  | ✓ | ✓ |
| Outcome | - Clinicians’ acceptability of implemented EMRs - Uptake/adoption of EMRs - Benefits of implemented EMRs for patient safety or care quality | Studies that did not report outcomes | ✓ | ✓ |
| Study type | - Peer-reviewed primary research with documented search strategy | - Protocols - Opinion pieces/editorials/letters - Conceptual/design/development papers | ✓ |  |
|  | - Reviews with documented search strategy only | - Reviews without documented search - Protocols - Opinion pieces and editorial letters - Conceptual/design/development papers |  | ✓ |

^a^ R1: Focused on reviews on clinician-facing EMRs generally, not mental health specific; results are termed “reviews” in this review.

^b^ R2: Focused primarily on studies on EMRs used in mental health settings by mental health clinicians; results are termed “primary studies” in this review.
