# Supplementary material for: Implementation of Electronic Medical Records in Mental Health Settings: Scoping Review
Source: JMIR Ment Health. 2021 Sep 7;8(9):e30564. doi: 10.2196/30564 (PMC8456340; doi:10.2196/30564)
Supplement: Multimedia Appendix 2 [file mental_v8i9e30564_app2.docx]

## Appendix 2

## Table 1. Quality assessment results of primary empirical mental health studies using a modified Hawker appraisal tool.^a^

| Reference | Abstract and title | Introduction and aims | Method and data | Sampling | Data analysis | Ethics and bias | Results | Transferability | Implications and usefulness | Overall rating /36 | Quality |
| --- | --- | --- | --- | --- | --- | --- | --- | --- | --- | --- | --- |
| Boyer et al (2010) [23] | 4 | 3 | 3 | 4 | 3 | 2 | 4 | 4 | 4 | 31 | High |
| Bruns et al (2018) [24] | 4 | 3 | 4 | 4 | 4 | 4 | 4 | 4 | 4 | 35 | High |
| Erlingsdóttier et al (2019) [25] | 4 | 4 | 2 | 2 | 3 | 2 | 3 | 2 | 3 | 25 | Medium |
| Golberstein et al (2018) [26] | 4 | 3 | 4 | 3 | 4 | 4 | 4 | 3 | 4 | 33 | High |
| Jeletelina et al (2018) [27] | 4 | 4 | 3 | 2 | 3 | 2 | 4 | 2 | 4 | 28 | Medium |
| Madden et al (2016) [28] | 3 | 3 | 4 | 4 | 4 | 2 | 4 | 4 | 3 | 31 | High |
| Martin et al (2018) [29] | 2 | 3 | 3 | 4 | 2 | 3 | 4 | 3 | 3 | 27 | Medium |
| Reyes-Portillo et al (2018) [30] | 4 | 4 | 4 | 2 | 3 | 4 | 4 | 3 | 4 | 32 | High |
| Riahi et al (2017) [31] | 4 | 4 | 4 | 4 | 4 | 2 | 4 | 4 | 4 | 34 | High |
| Skelton et al (2019) [33] | 3 | 2 | 3 | 2 | 2 | 2 | 4 | 2 | 4 | 24 | Medium |
| Stanhope et al (2019) [34] | 4 | 3 | 3 | 2 | 3 | 2 | 4 | 2 | 4 | 27 | Medium |
| Ser et al (2014) [32] | 4 | 4 | 4 | 4 | 4 | 4 | 4 | 4 | 4 | 36 | High |

**^a^** Scoring explanation: 4 = Good, 3 = Fair, 2 = Poor, 1 = Very poor.

**Table 2.** Quality appraisal results of reviews in general health settings using the Joanna Briggs Institute Critical Appraisal Checklist for Systematic Reviews and Research Syntheses.

| Reference | Is the review question clearly and explicitly stated? | Were the inclusion criteria appropriate for the review question? | Was the search strategy appropriate? | Were the sources and resources used to search for studies adequate? | Were the criteria for appraising studies appropriate? | Was critical appraisal conducted by two or more reviewers independently? | Were there methods to minimize errors in data extraction? | Were the methods used to combine studies appropriate? | Was the likelihood of publication bias assessed? | Were recommendations for policy and practice supported by the reported data? | Were the specific directives for new research appropriate? | Number of Yes categories (0-11) |
| --- | --- | --- | --- | --- | --- | --- | --- | --- | --- | --- | --- | --- |
| Bauman et al (2018) [35] | Yes | Yes | Yes | Yes | Unclear | Unclear | Unclear | Yes | Yes | Yes | Yes | 8 |
| Boonstra et al (2010) [36] | Yes | Yes | Yes | Yes | Unclear | Unclear | Yes | Yes | No | Yes | N/A | 7 |
| Castillo et al (2010 )[37] | Yes | Yes | Yes | Yes | Unclear | Unclear | Yes | Yes | No | Yes | Yes | 8 |
| Delardes et al (2020) [38] | Yes | Yes | Yes | Yes | Yes | Yes | Unclear | Yes | Unclear | Yes | Yes | 9 |
| Gephart et al (2015) [39] | No | Yes | Yes | Yes | Unclear | Unclear | Unclear | Unclear | Unclear | Yes | Yes | 5 |
| Goldstein et al (2014) [40] | Yes | Yes | Yes | Yes | Yes | Yes | Yes | Yes | No | Yes | Yes | 10 |
| Goldzweig et al (2015) [41] | Yes | Yes | Yes | No | Yes | Unclear | Yes | Yes | Yes | Yes | Yes | 9 |
| Lau et al (2012) [42] | Yes | Yes | Yes | Yes | Unclear | Unclear | Yes | Yes | No | Yes | Yes | 8 |
| Meiβner et al (2014) [43] | Yes | Yes | Yes | Yes | Unclear | Unclear | Unclear | Yes | N/A | Yes | Yes | 7 |
| Nguyen et al (2014) [44] | No | Unclear | Yes | Yes | Unclear | Unclear | Yes | Yes | No | Yes | Yes | 6 |
| Strudwick et al (2015) [45] | Yes | Yes | Yes | Yes | Unclear | Unclear | Unclear | Unclear | No | Yes | Yes | 6 |
